# Crystal structure of an inactive variant of the quorum-sensing master regulator HapR from the protease-deficient non-O1, non-O139 *Vibrio cholerae* strain V2

**DOI:** 10.1107/S2053230X18006519

**Published:** 2018-05-17

**Authors:** Justin Cruite, Patrick Succo, Saumya Raychaudhuri, F. Jon Kull

**Affiliations:** a Department of Biochemistry, Geisel School of Medicine, Dartmouth College, Hanover, New Hampshire, USA; b College of Veterinary Medicine and Biomedical Sciences, Colorado State University, Fort Collins, Colorado, USA; c Institute of Microbial Technology, Chandigarh, Council of Scientific and Industrial Research, Chandigarh 160 036, India; d Department of Chemistry, Dartmouth College, Hanover, New Hampshire, USA

**Keywords:** HapR, quorum sensing, *Vibrio cholerae*, TetR transcriptional regulator

## Abstract

HapR is a TetR-family transcriptional regulator that controls quorum sensing in *Vibrio cholerae*, the causative agent of cholera. HapR regulates the expression of hemagglutinin protease, virulence and biofilm genes. The crystal structure of wild-type HapR from *V. cholerae* strain O1 El Tor C6706 has previously been solved. In this study, the structure of a DNA-binding-deficient variant of HapR (HapR_V2_) derived from the protease-deficient *V. cholerae* serotype O37 strain V2 is reported. The structure reveals no structural differences compared with wild-type HapR. However, structural alignment of HapR_V2_ with the TetR-family member QacR in complex with its operator DNA suggests that the aspartate residue located between the regulatory and DNA-binding domains may clash with and electrostatically repel the phosphate backbone of DNA to prevent binding.

## Abstract 2

The crystal structure of an inactive variant of the the quorum-sensing master transcription regulator HapR from *Vibrio cholerae* suggests that its inactivity is owing to steric clashes and charge repulsion with the phosphate backbone of DNA.

## 1. Introduction   

The acute diarrheal disease cholera is caused by ingesting food or water contaminated with the Gram-negative bacterium *Vibrio cholerae*. The world is currently experiencing a seventh global cholera pandemic, which began in 1961. In 2015, 42 countries reported 172 454 cases and 1304 deaths. However, it is estimated that the actual number of cholera cases is between 1.3 and 4 million per year and that 21 000–143 000 die from the disease each year worldwide (World Health Organization, 2016 ▸).

Toxigenic *V. cholerae* causes disease by producing two primary virulence factors: cholera toxin (CT) and the toxin-coregulated pilus (TCP) (Taylor *et al.*, 1987 ▸). The expression of these virulence factors is controlled by a network of transcriptional regulators that is initiated when AphA cooperates with AphB to activate the expression of TcpPH (Skorupski & Taylor, 1999 ▸; DiRita *et al.*, 1991 ▸; Kovacikova & Skorupski, 1999 ▸; Kovacikova *et al.*, 2004 ▸, 2010 ▸). Together with ToxRS, TcpPH induces the expression of ToxT, which directly activates the expression of CT and TCP (Miller *et al.*, 1987 ▸; Higgins & DiRita, 1994 ▸; Häse & Mekalanos, 1998 ▸; Goss *et al.*, 2010 ▸).

Like many bacteria, *V. cholerae* uses quorum sensing to regulate gene expression in response to an increase in cell density (Miller & Bassler, 2001 ▸). *V. cholerae* secretes the autoinducers CAI-1 and AI-2 (Miller *et al.*, 2002 ▸). As the cell density increases, the extracellular concentration of these autoinducers also increases. At low cell density, the response regulator LuxO is phosphorylated by the sensor kinases CqsS and LuxP (Higgins *et al.*, 2007 ▸). Phosphorylated LuxO activates the transcription of four small regulatory RNAs that inhibit the translation of the quorum-sensing master regulator HapR (Lenz *et al.*, 2004 ▸). At high cell density, CqsA and LuxS detect CAI-1 and AI-2, respectively, which leads to the dephos­phorylation of LuxO and the production of HapR. HapR regulates the expression of a large number of genes in *V. cholerae*: it activates the expression of hemagglutinin protease (Silva & Benitez, 2004 ▸), enhances the stress response (Joelsson *et al.*, 2007 ▸), enhances predation-driven persistence (Matz *et al.*, 2005 ▸), promotes chitin-induced competence (Meibom *et al.*, 2005 ▸), regulates *hcp* expression (Ishikawa *et al.*, 2009 ▸), negatively regulates virulence-gene expression by repressing the expression of AphA (Zhu *et al.*, 2002 ▸; Kovacikova & Skorupski, 2002 ▸; Lin *et al.*, 2007 ▸) and represses biofilm formation by repressing *vpsR* (Hammer & Bassler, 2003 ▸ *)*.

HapR is a member of the TetR family of transcriptional regulators (Jobling & Holmes, 1997 ▸). The crystal structure of wild-type HapR revealed that the protein is entirely α-helical and contains an N-terminal helix–turn–helix DNA-binding domain and a C-terminal dimerization/regulatory domain typical of TetR-family members (De Silva *et al.*, 2007 ▸). Within each regulatory domain is an amphipathic cavity that may serve as a binding pocket for a yet to be identified ligand.

A surprising number of epidemic-causing O1/O139 strains as well as non-O1/non-O139 strains of *V. cholerae* isolated globally have been found to have dysfunctional quorum-sensing systems (Joelsson *et al.*, 2006 ▸; Talyzina *et al.*, 2009 ▸; Wang *et al.*, 2011 ▸). Of these, several have mutations in *hapR*. The classical strain O395 and the El Tor strain N16961 both have frameshift mutations that place a premature stop codon upstream of the C-terminal dimerization domain of HapR. A portion of the dimerization domain of HapR is deleted in strain SG1. Strains MO10, 857 and MAK757 have one, two and seven point mutations in *hapR*, respectively. Strain MDO14-T completely lacks *hapR*.

The protease-deficient *V. cholerae* serotype O37 strain V2 was isolated in Calcutta, India in 1989. Strain V2 was recently found to contain a glycine-to-aspartate substitution at position 39 within the hinge region between the DNA-binding and dimerization domains of HapR (HapR_V2_; Dongre *et al.*, 2011 ▸). In their study, Dongre and coworkers showed by EMSA that HapR_V2_ was defective in DNA-binding activity. Size-exclusion chromatography and circular dichroism revealed no significant structural differences between normal and variant HapR. Furthermore, Guinier analysis and indirect Fourier transformation of small-angle X-ray scattering (SAXS) indicated only a slight difference in shape. However, structural reconstruction using the SAXS data suggested that the arrangement of the DNA-binding domains of the variant HapR was altered. To gain further insight into the functional role of the hinge region of HapR and the structural consequences of a substitution of aspartate for glycine at position 39, we determined the crystal structure of HapR_V2_ to a resolution of 2.1 Å. The structure suggests that the aspartate located in the hinge region of HapR_V2_ would sterically clash with and electrostatically repel DNA, preventing the binding and the regulation of genes controlled by the quorum-sensing system of *V. cholerae*.

## 2. Materials and methods   

### 2.1. Expression and purification   

The ORF for HapR_V2_ was cloned into pET-15b to generate thrombin-cleavable N-terminally six-His-tagged HapR_V2_, as described previously by Dongre *et al.* (2011 ▸). HapR_V2_ was expressed in *Escherichia coli* BL21(DE3) cells induced by autoinduction in ZYM-5052 medium overnight at 20°C (Studier, 2005 ▸). The cells were lysed in 20 m*M* Tris–HCl pH 8, 100 m*M* NaCl by sonication at 4°C and centrifuged at 120 000*g* for 30 min. The supernatant was filtered using a 0.45 µm filter and loaded onto a GE HisTrap FF column using an ÄKTAexplorer FPLC system. The column was eluted with a linear gradient of 40–500 m*M* imidazole and a single peak was collected. The protein was further purified using a GE SP FF cation-exchange column and a Superdex S75 16/600 size-exclusion column.

### 2.2. Crystallization   

Purified HapR_V2_ was concentrated to 5 mg ml^−1^ using Amicon Ultra centrifugal filter units. Crystallization conditions were screened by the sitting-drop vapor-diffusion method. Diffraction-quality single crystals were obtained by mixing equal volumes of protein solution and 0.1 *M* MES pH 6.5, 15% PEG 20K. Crystals appeared after 6 d. Crystallization solution supplemented with 35% ethylene glycol was used as a cryoprotectant and crystals were flash-cooled in liquid nitrogen.

### 2.3. Data collection and processing   

X-ray diffraction data were collected on beamline X6A at the National Synchrotron Light Source (NSLS), Brookhaven National Laboratory, Upton, New York, USA. A 2.1 Å resolution data set of 360 frames with an oscillation range of 0.5° was collected at a wavelength of 1.000 Å with 15 s exposures at 100 K. The crystal-to-detector distance was 220 mm. The data set was indexed, integrated, scaled and merged using *XDS* (Kabsch, 1993 ▸). Data-collection statistics are shown in Table 1 ▸.

### 2.4. Structure solution and refinement   

The reflection file was converted and *R*_free_ flags were set (7.89% of unique reflections) using the *PHENIX* reflection-file editor (Adams *et al.*, 2002 ▸). The Matthews coefficient was calculated and it was determined that the asymmetric unit contained a single dimer of HapR_V2_. The structure of HapR_V2_ was solved by molecular replacement with *PHENIX Phaser-MR* (McCoy *et al.*, 2007 ▸) using wild-type HapR (PDB entry 2pbx; De Silva *et al.*, 2007 ▸) as the search model. Multiple rounds of refinement were carried out using *Coot* and *phenix.refine* (Emsley & Cowtan, 2004 ▸; Afonine *et al.*, 2012 ▸). Refinement statistics are shown in Table 1 ▸.

### 2.5. Structural alignments and modeling   

All structural alignments, modeling and distance measurements were performed with the *PyMOL* molecular-graphics system (DeLano, 2002 ▸).

## 3. Results and discussion   

### 3.1. Structure of HapR_V2_   

**Figure 1:**
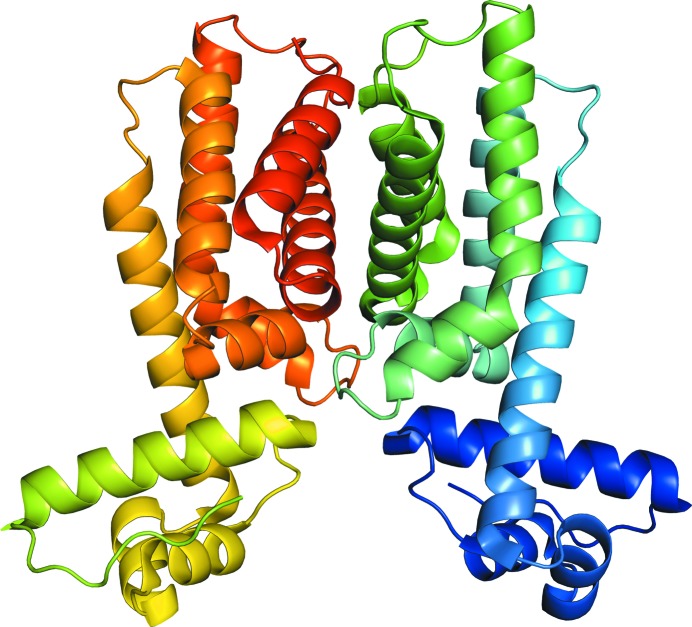
(*a*) The asymmetric unit of HapR_V2_ (PDB entry 6d7r). The individual subunits of the dimer are colored from the N-terminus to the C-­terminus in dark blue to green (right) and light green to red (left).

The crystal structure of HapR_V2_ was refined to a resolution of 2.1 Å (Fig. 1 ▸). There is one homodimer of HapR_V2_ in the asymmetric unit. The two subunits in each dimer are related by twofold noncrystallographic symmetry. As in wild-type HapR, the structure of HapR_V2_ is entirely α-helical. The first three helices of each monomer form a helix–turn–helix DNA-binding domain. Helices 4–9 form the dimerization/regulatory domain.

### 3.2. Alignment of HapR_V2_ with wild-type HapR   

**Figure 2:**
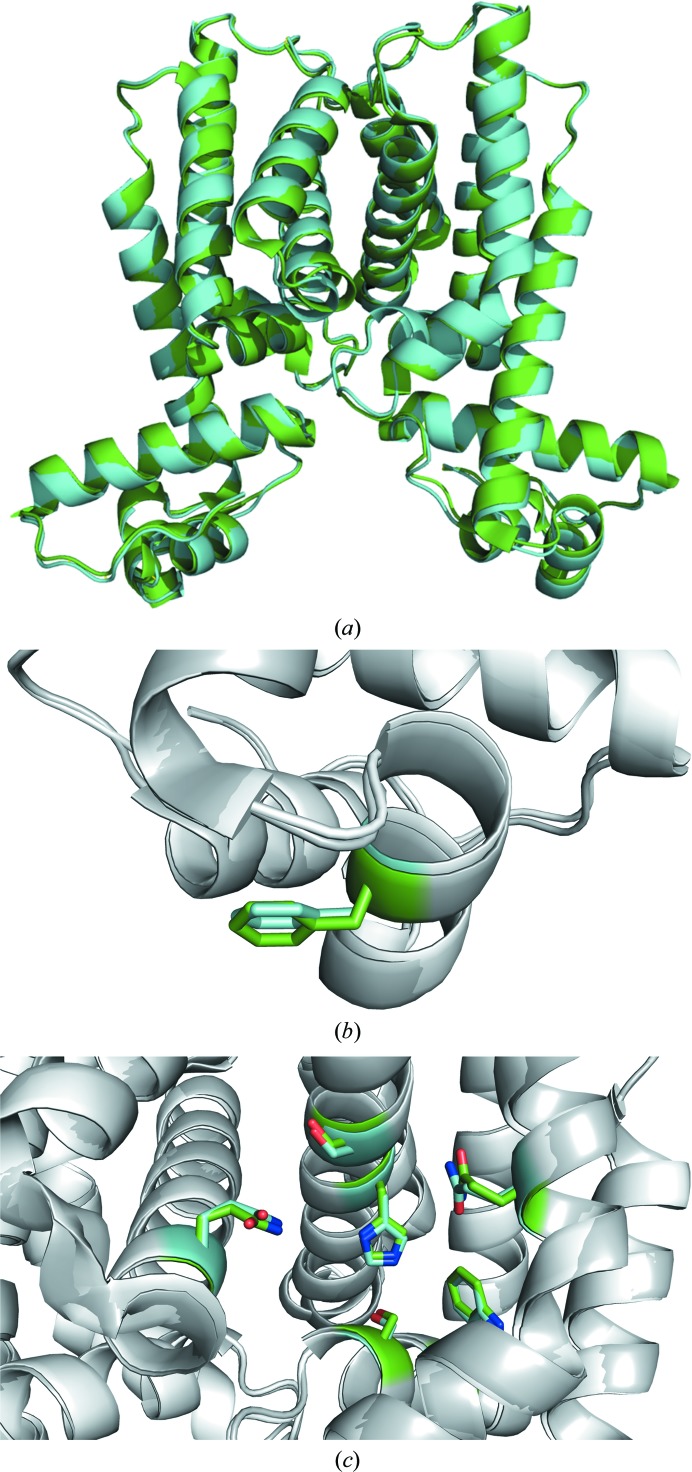
(*a*) Superposition of HapR_V2_ (green) with the previously determined wild-type structure (cyan; PDB entry 2pbx). (*b*) Alignment of Phe55 of HapR_V2_ (gray/green) and wild-type HapR (white/cyan). (*c*) Alignment of residues within the putative ligand-binding pockets of HapR_V2_ and wild-type HapR.

Although previous structural reconstructions using small-angle X-ray scattering data have suggested that the DNA-binding domain of HapR_V2_ adopts an altered conformation relative to the wild type, the crystal structure of HapR_V2_ reveals that there are no significant structural differences between HapR_V2_ and wild-type HapR (Fig. 2 ▸ *a*). Alignment of the HapR_V2_ dimer with wild-type HapR results in an r.m.s.d. of 0.448 Å for 381 C^α^ atoms. Phe55, which has been shown to be necessary for DNA binding (De Silva *et al.*, 2007 ▸), is in an identical position to that in wild-type HapR (Fig. 2 ▸ *b*). Furthermore, residues within the putative ligand-binding pocket of HapR_V2_ are in the same positions as in the wild type (Fig. 2 ▸ *c*).

### 3.3. Alignment of HapR_V2_ with SmcR   

**Figure 3:**
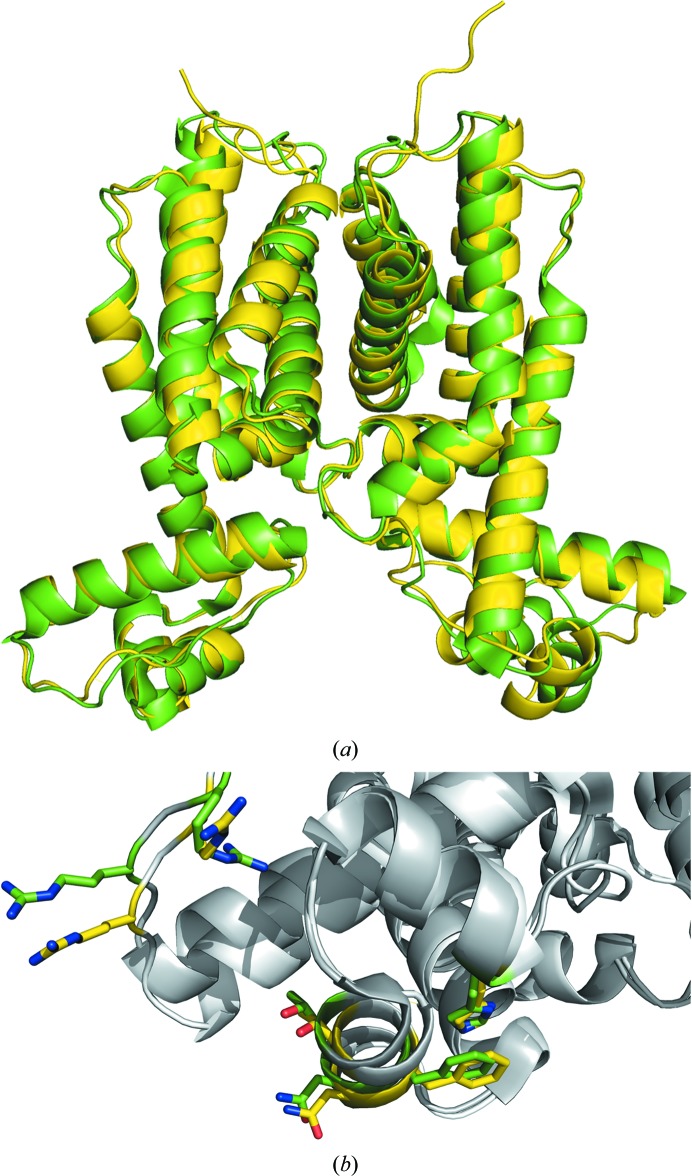
(*a*) Superposition of the structure of HapR_V2_ (green) with that of SmcR (yellow; PDB entry 3kz9; Kim *et al.*, 2010 ▸) from *V. vulnificus*. (*b*) Alignment of residues in the DNA-binding domains of HapR_V2_ (gray/green) and SmcR (white/yellow).

SmcR is a homolog of HapR that regulates quorum sensing in *V. vulnificus* (Kim *et al.*, 2010 ▸). Alignment of the HapR_V2_ dimer with SmcR results in an r.m.s.d. of 0.78 Å for 308 C^α^ atoms (Fig. 3 ▸ *a*). The superposition revealed a close alignment of residues Arg10, Arg12, His40, Thr53, Phe55 and Asn56 of HapR_V2_ with Arg9, Arg11, His39, Thr52, Phe54 and Asn55 of SmcR, all of which were shown to be necessary for SmcR to bind DNA (Fig. 3 ▸ *b*).

### 3.4. Alignment of HapR_V2_ with the QacR–DNA complex   

**Figure 4:**
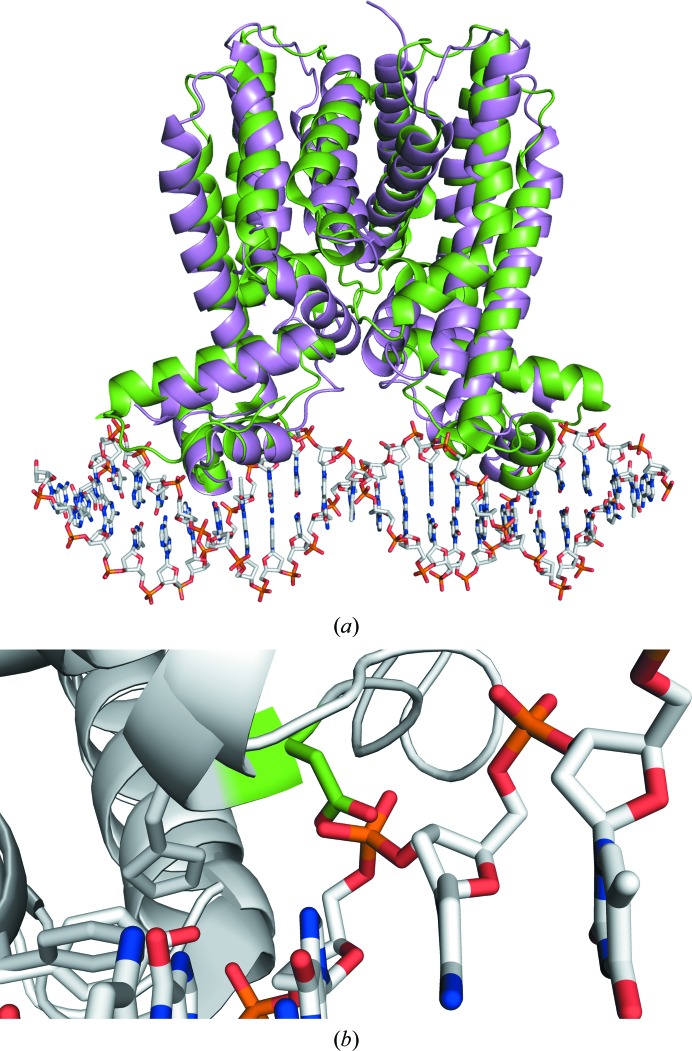
(*a*) Superposition of the structure of HapR_V2_ (green) with that of QacR–DNA (violet) from *S. aureus* (PDB entry 1jt0; Schumacher *et al.*, 2002 ▸). The r.m.s.d. for 284 atoms is 2.3 Å. (*b*) The position of Asp39 (green) of HapR_V2_ (gray) when aligned with the QacR–DNA structure (white).

In order to gain further insight into the reason that HapR_V2_ is unable to bind DNA, the HapR_V2_ structure was aligned with that of the *Staphylococcus aureus* multidrug-binding transcriptional repressor QacR in complex with its operator DNA (Fig. 4 ▸ *a*). HapR_V2_ aligns with QacR with an r.m.s.d. of 2.3 Å for 284 C^α^ atoms. The alignment positions the DNA-binding helices within adjacent major grooves of the DNA double helix. The C^α^ atoms of Phe55 in each subunit of the HapR_V2_ dimer are 39.7 Å apart, which is only 2.8 Å further apart than the C^α^ atoms of Tyr40 at the analogous positions in QacR. Interestingly, the positioning of HapR_V2_ on DNA revealed that the carboxyl side chain of Asp39 may both sterically clash with and electrostatically repel the phosphate backbone of DNA, possibly explaining the inability of this variant to bind DNA (Fig. 4 ▸ *b*).

### 3.5. Electrostatic surface potential of HapR_V2_ *versus* the wild type   

**Figure 5:**
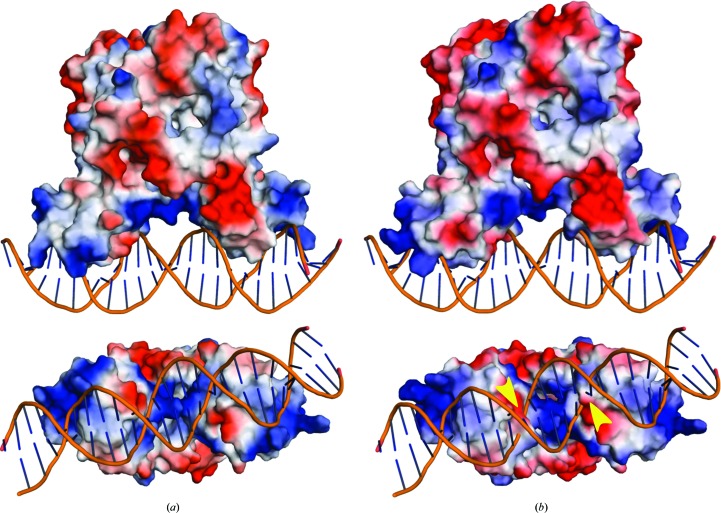
Electrostatic surfaces of wild-type HapR (*a*) and HapR_V2_ (*b*) positioned on DNA as aligned with the QacR–DNA structure. Positively charged surface is colored blue; negatively charged surface is colored red. Yellow arrows indicate where the side chain of Asp39 in HapR_V2_ would overlap with the phosphate backbone of DNA.

A comparison of the electrostatic surface potential of HapR_V2_ with that of wild-type HapR revealed only subtle differences in the positions of charged residues (Fig. 5 ▸). However, the electrostatic surface of HapR_V2_ positioned on DNA by alignment with QacR shows that the negatively charged surface of Asp39 would overlap with the DNA backbone if bound (Fig. 5 ▸ *b*).

## 4. Conclusion   

Given the number of *V. cholerae* isolates that have been found with nonfunctional quorum-sensing systems, it can be assumed that the loss confers some advantage to the bacterium. The classical pandemic serotype O1 strain O395 and the El Tor strain N16961 both have mutations in the quorum-sensing master regulator HapR. The nonfunctional HapR from *V. cholerae* serotype O37 strain V2, isolated in Calcutta, India, bears a substitution of aspartate for glycine at position 39, which is within the hinge region between the DNA-binding and regulatory domains. The crystal structure of HapR_V2_ suggests that the carboxylate side chain of Asp39 would clash with and electrostatically repel the phosphate backbone of DNA, preventing DNA binding by HapR_V2_ and therefore the regulation of quorum-controlled genes.

## Supplementary Material

PDB reference: HapR, G39D mutant, 5l0x

## Figures and Tables

**Table 1:** Data-collection and refinement statistics for the crystal structure of HapR_V2_ (PDB entry 6d7r) Values in parentheses are for the highest resolution shell.

Data collection	
Space group	*P*2_1_2_1_2_1_
Mosaicity (°)	0.200
Resolution (Å)	19.428–2.100 (2.175–2.100)
Wavelength (Å)	1.0000
Temperature (K)	100
Observed reflections	182643
Unique reflections	25245 (2496)
〈*I*/σ(*I*)〉	19.04 (2.08)
Completeness (%)	99.8 (100)
Multiplicity	7.23
*R*_meas_ (%)	8.7 (121.7)
CC_1/2_	1.00 (0.745)
Refinement	
Resolution (Å)	19.428–2.100
Reflections (working/test)	25244 (2495)
*R*_work_/*R*_free_ (%)	20.25/26.80
No. of atoms	
Protein	3246
Water	79
Model quality	
R.m.s. deviations	
Bond lengths (Å)	0.008
Bond angles (°)	0.93
Wilson *B* factor (Å^2^)	36.26
Coordinate error (maximum likelihood) (Å)	0.31
Ramachandran plot	
Favored (%)	96.92
Allowed (%)	2.82
Outliers (%)	0.26
Rotamer outliers (%)	0.00
Clashscore	10.5
